# Convolutional neural network for breast cancer diagnosis using diffuse optical tomography

**DOI:** 10.1186/s42492-019-0012-y

**Published:** 2019-05-08

**Authors:** Qiwen Xu, Xin Wang, Huabei Jiang

**Affiliations:** 1 School of Electronic Science and Engineering, University of Electronic Science and Technology of China, Chengdu, 611731 China; 2 Center for Information in Medicine, University of Electronic Science and Technology of China, Chengdu, 611731 China; 3 Department of Medical Engineering, University of South Florida, Tampa, FL 33620 USA

**Keywords:** Diffuse optical tomography, Breast cancer, Convolutional neural network, Machine learning, Image classification

## Abstract

We have developed a computer-aided diagnosis system based on a convolutional neural network that aims to classify breast mass lesions in optical tomographic images obtained using a diffuse optical tomography system, which is suitable for repeated measurements in mass screening. Sixty-three optical tomographic images were collected from women with dense breasts, and a dataset of 1260 2D gray scale images sliced from these 3D images was built. After image preprocessing and normalization, we tested the network on this dataset and obtained 0.80 specificity, 0.95 sensitivity, 90.2% accuracy, and 0.94 area under the receiver operating characteristic curve (AUC). Furthermore, a data augmentation method was implemented to alleviate the imbalance between benign and malignant samples in the dataset. The sensitivity, specificity, accuracy, and AUC of the classification on the augmented dataset were 0.88, 0.96, 93.3%, and 0.95, respectively.

## Introduction

Breast cancer is the most common cancer among women. To reduce the mortality of breast cancer, early detection and an accurate diagnosis are important [1]. Thus, a promotable and sensitive detection technology with efficient statistical analysis methods are necessary for mass screening for breast cancer.

There are currently several clinical methods for breast cancer detection including X-ray mammography, magnetic resonance imaging (MRI), and ultrasound. X-ray mammography is the most commonly used method for breast detection; however, it may cause damage owing to ionization, making it unsuitable for repeated mass screening measurements [2, 3]. MRI can offer excellent images of breast tissue with higher sensitivities; however, MRI incurs high costs, low specificities, and is not very convenient, which greatly limits its application [4–6]. As a relatively inexpensive and nonionizing radiation method, ultrasound can differentiate between benign and malignant masses; however, it has a low sensitivity and is highly dependent on the skill of the technician [4, 7]. Other methods, such as photoacoustic imaging, are also emerging as diagnostic techniques for breast cancer detection [8–10]. Diffuse optical tomography (DOT) is an emerging method for breast tumor diagnosis that provides optical properties of breast tissue correlating to the tumor’s physiological signatures [11, 12]. As an imaging modality with endogenous contrast, DOT has the potential to overcome the limitations of the abovementioned modalities, particularly when screening dense breasts, in terms of safety, cost, portability, sensitivity, and specificity. DOT also allows for improved discrimination between malignant and benign lesions, making it a competitive mass screening method for breast cancer.

Thus far, several studies have applied computer-aided diagnosis (CAD) techniques to breast cancer detection; these techniques include the use of artificial neural networks [13–15], fuzzy logic [16, 17], Bayesian networks [18, 19], decision trees [20, 21], and k-means clustering [22, 23]. However, few researchers have implemented CAD methods with DOT to diagnose breast cancer. A study analyzed the absorption, scattering, and refractive index images obtained by a phase-contrast diffuse optical tomography system and demonstrated the sensitivity and specificity to be 0.82 and 0.92, respectively, with a support vector machine (SVM) classifier [24]. Another study proposed a semi-automatic detection method of malignant breast lesions in DOT images using logistic regression of three optically measured physiological parameters; this method had an average sensitivity and specificity of 0.89 and 0.89, respectively [25].

Recently, convolutional neural networks (CNNs) have been proven to work well in the differentiation between benign and malignant breast lesions [26–30]. Compared with traditional methods, CNNs reduce the steps involved in image feature extraction; alternatively, they feed image data directly into the network that can learn discriminative features automatically. CNN architecture is particularly adapted to take advantage of the 2D structure of the input image.

In this work, a dataset of DOT breast images with 63 biopsy-confirmed tumor-bearing dense breasts were used. Dense breasts are common among young women and cannot be examined by existing mammography imaging systems [31]. All images were collected from a DOT system and reconstructed with a finite element algorithm [32]. In our previous work, an SVM classifier was used to classify breast tumors and achieved an accuracy of 71.7%. This study develops an optimized CNN adapted to the characteristics of diffusion tomography images; this proposed CNN shows a high accuracy on the breast cancer dataset.

## Methods

**Fig. 1:**
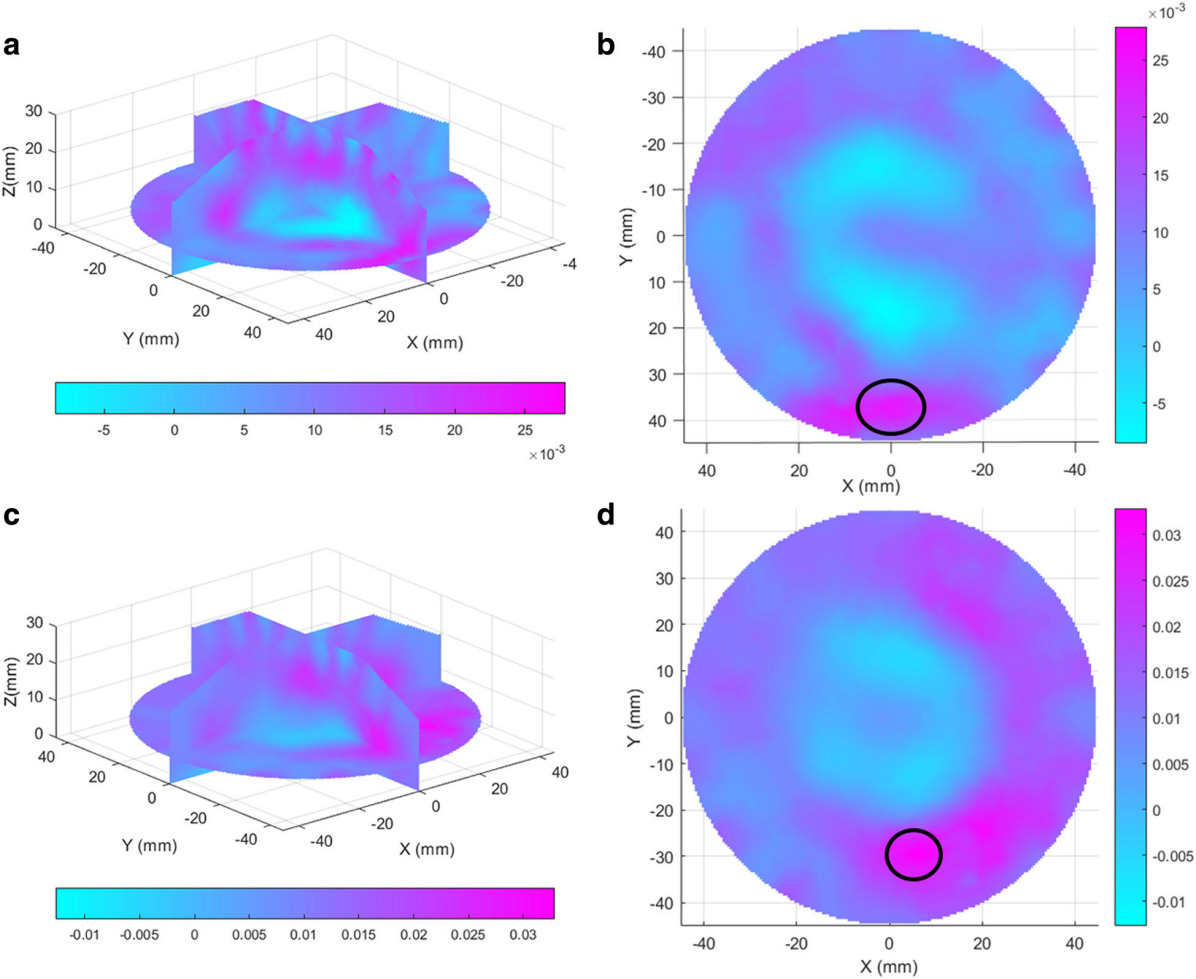
Examples of diffuse optical tomography breast images. **a** 3D image of a benign breast tumor with a size of 3.0*1.3*1.3**, b** 2D image sliced from Fig. 1a**, c** 3D image of a malignant breast tumor with a size of 2.4*1.3*1.2**, d** 2D image sliced from Fig. 1c

CNNs require a large amount of training data to achieve high accuracies. However, it is difficult to build a comprehensive dataset with a large number of breast tumor images. The collected 3D images are gray scale images with a size of 91 × 91 × 31. Figure 1 illustrates DOT images of a benign tumor-bearing breast and a malignant tumor-bearing breast. It is difficult to distinguish tumor and normal tissue from the images owing to the high breast density. We found that most tumors are distributed in the range of 6 to 25 on the Z-axis of the 3D image by matching the biopsy result to the image and chose to slice images from this range. A total of 20 slices in each 3D image was used for this dataset. The total dataset consisted of 1260 2D images, obtained from slicing the 63 3D images. Each 2D image is resized to a size of 32 × 32 and normalized for CNN input. The dataset was randomly divided into training and test groups that contained 75% and 25% of the data, respectively, as shown in Table 1.

**Table 1:** 2D diffuse optical tomography image dataset and its division into training and test sets

	Benign	Malignant
Training set	645	300
Test set	215	100

**Fig. 2:**
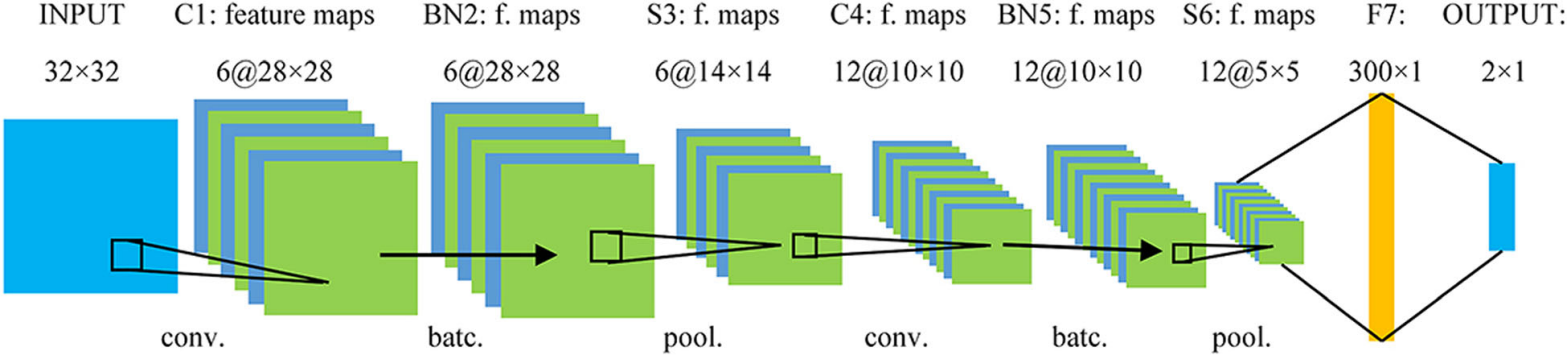
Architecture of the convolutional neural network

A CNN-based CAD method was proposed to classify breast mass lesions. The architecture of our network is summarized in Fig. 2. It contains five learned layers including two convolutional, two batch normalization, and one fully-connected layer. The input of the network is a 32 × 32 Gy scale image. The first convolutional layer, C1, filters the 32 × 32 input gray scale image with six kernels of size 5 × 5 with a stride of 1 pixel. The second convolutional layer, C4, takes the normalized and pooled output of the first convolutional layer as its input and filters it with 12 kernels of size 5 × 5.

Each convolutional layer is followed by a batch normalization layer, which aims to stabilize the distributions of layer inputs by controlling the mean and variance [33]. It adds two extra parameters per activation to preserve the representation ability of the network. Because of batch normalization, the network can tolerate increased training rates and often does not require dropouts for regularization. The normalized results then pass through a sigmoid nonlinear function as an output for these layers.

Pooling layers perform local averaging and subsampling, thereby reducing the resolution of the feature map and the sensitivity of the output to shifts and distortions. Pooling layers have a filter size of 2 × 2 pixels and output the average value offour inputs in each local region.

A simple logistic classifier is used in the final layer of the network. Malignant and benign are represented with 2-bit one-hot encoding as (1,0) and (0,1) in the output layer. The mini-batch stochastic gradient descent momentum is used for training. We also adopted an early stopping strategy to monitor the test misclassification rate (MCR), where training is terminated if no progress is noted on the test set. Optimum results were obtained by tuning the hyper parameters through an extensive set of trial and error experiments; finally, we used a learning rate of 0.3, mini-batch size of 83, and maximum epochs of 500.

Owing to the imbalance between the number of benign and malignant samples in the dataset, we use two groups of sets for training. The first set is the original training set and includes 945 images, and the second set, which includes 1245 images, is the augmented version of the first group where the number of malignant images was doubled by flipping the images. The distribution of the augmented dataset is given in Table 2. Training images are used to train the network and the remaining ones are used for testing. The network predictions and the biopsy results were compared using a receiver operating characteristic (ROC) analysis. Differences in performance are evaluated by computing the area under the ROC curves (AUC).

**Table 2:** Augmented dataset and its division into training and test sets

	Benign	Malignant
Training set	645	600
Test set	215	100

## Results

**Fig. 3:**
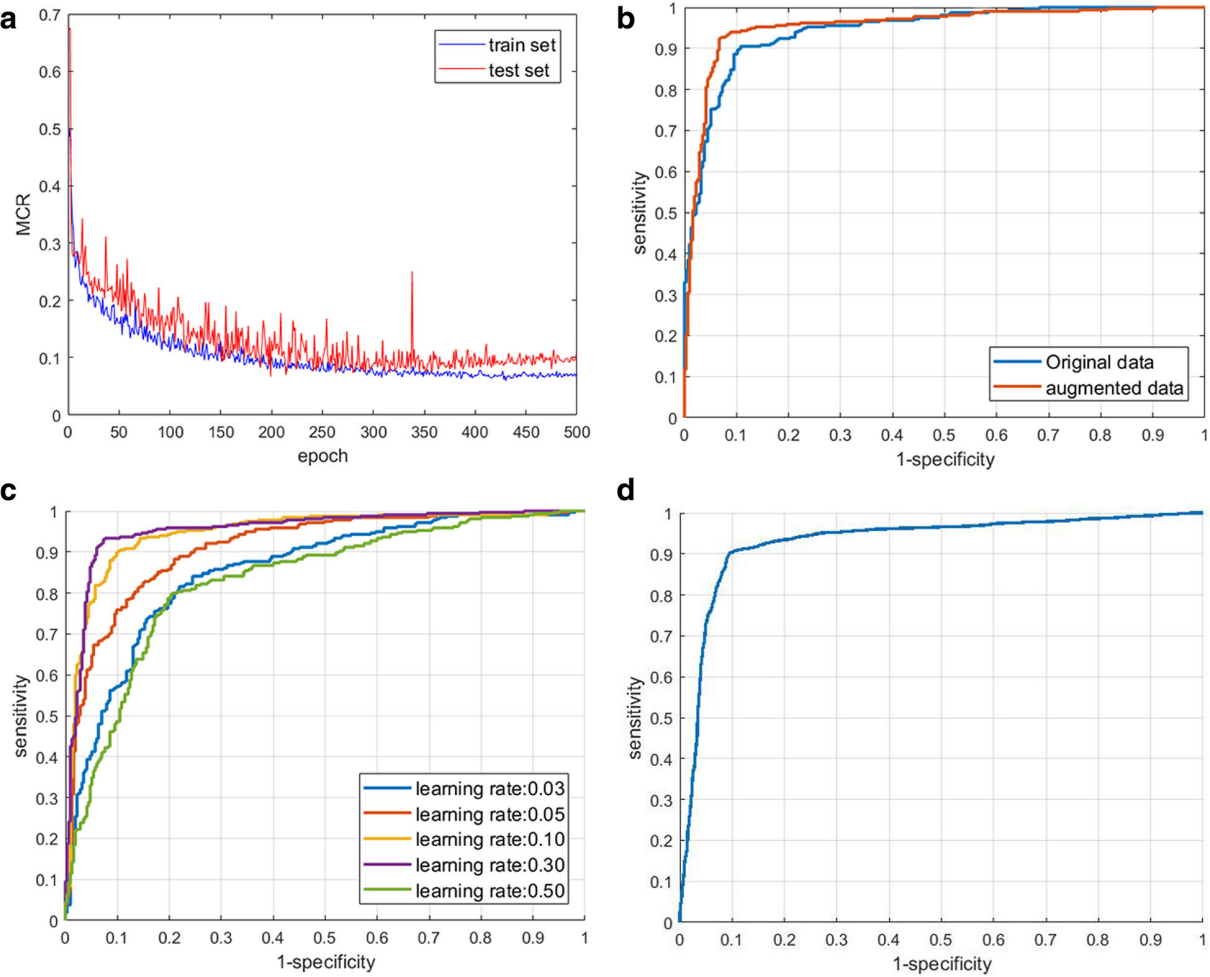
**a** Misclassification rate curve of training set and testing set during training, **b** Receiver operating characteristic (ROC) comparison of original data and augmented data, **c** ROC comparison of convolutional neural network (CNN) with different learning rate, **d** ROC of CNN evaluated by a 10-fold cross-validation

Our results on the DOT breast dataset are summarized in Table 3. Sensitivity and specificity are statistical indexes of the performance of a binary classification test; they measure the proportion of correctly identified positives and negatives. Our network achieves a test accuracy rate of 90.2%, a specificity of 0.80, and a sensitivity of 0.95 compared with the original data. More satisfactory results are obtained when the network is trained on the augmented data, with a test accuracy rate of 93.3%, a specificity of 0.88, and a sensitivity of 0.96; this is the best performance achieved in this study. The MCR curve of the training and test sets during training is shown in Fig. 3a. The test set was checked during training to monitor progress and to apply the early stopping strategy when a plateau was reached. The proposed network can be trained faster and requires fewer training steps (four times) to achieve the same accuracy compared with a simple CNN without batch normalization. The minimum test MCR was achieved after approximately 200 epochs during training. There was no obvious overfitting when data augmentation and batch normalization are used. The ROC curve comparing the performance of the network trained on the original and augmented data is shown in Fig. 3b. The AUC is 0.94 for the network trained on the original data and 0.95 for one trained on the augmented data.

**Table 3:** Comparison summary of the convolutional neural network performance on original and augmented data

	Accuracy	Specificity	Sensitivity	Area under curve
Original data	90.2%	0.80	0.95	0.94
Augmented data	93.3%	0.88	0.96	0.95

The network is also trained in five groups of learning rates to obtain the best performance. The learning rate defines the speed at which the weight is updated on each epoch in the neural network. A network may learn fast but may be unstable and exhibit a very high learning rate. Conversely, a network that learns slowly may be easily susceptible local minimum trapping. Comparing the ROC curves in Fig. 3c, the suitable learning rate is determined to be 0.3.

During the study, we repeated the experiment several times and found that the test results show subtle differences. Thus, we trained and evaluated the CNN using a 10-fold cross-validation for robust testing. The ROC curve obtained is shown in Fig. 3d and the average AUC is found to be 0.93 (±0.03). This gives a sensitivity of 0.79 and a specificity of 0.97. It indicates that the CNN model is robust and is reliable for breast tumor classification. The average test accuracy rate reaches 91% with a standard deviation of 1.66%; this is a promising result achieved with little sample data.

## Conclusions

Our results in this work show that it is possible to achieve a satisfactory result on breast cancer diagnosis using a CNN trained with DOT breast images. A DOT breast dataset is built; it includes 63 patient samples with malignant or benign tumors, for a total of 1260 2D gray scale images. Although it is an arduous task to detect a dense breast with a small set of data, the proposed 8-layer CNN has a good capability of classifying image patterns while achieving a specificity and sensitivity of 0.88 and 0.96, respectively. This technique has the potential to assist radiologists in diagnosing breast cancer and improving the diagnostic rate that furthermore promotes mass screening for breast cancer.

In future studies, more samples will be collected to improve generalization as the accuracy can be further improved with a larger dataset. It is important to carefully select a better convolutional network architecture and optimization algorithm. We would like to train a 3D CNN on 3D data, where spatial structures can provide information that may be missing or is far less obvious in 2D images. Unsupervised pre-training is also being considered for our diagnosis method in the future.

The images used in this study are reconstructed with optical absorption distribution of tissue; however, DOT not only provides the optical absorption coefficients but can also determine the optical scattering coefficients. As a functional optical imaging system, DOT can quantify the tumor hemoglobin concentration and blood oxygen saturation; these are directly related to tumor angiogenesis. The focus of our next research study will be how to make full use of this information.
